# Cardiovascular Risk Management With Liaison Critical Path in Japan: Its Effects on Implementation of Evidence-Based Prevention in Practice

**DOI:** 10.4021/jocmr845w

**Published:** 2012-03-23

**Authors:** Morihiro Matsuda, Manami Akizuki, Orie Nishimoto, Kei Nakamoto, Hirohiko Nishiyama, Ritsu Tamura, Toshiharu Kawamoto

**Affiliations:** a Department of Cardiology, National Hospital Organization, Kure Medical Center, 3-1 Aoyama-cho, Kure, Hiroshima 737-0023, Japan; b Department of Internal Medicine, National Hospital Organization, Kure Medical Center, 3-1 Aoyama-cho, Kure, Hiroshima 737-0023, Japan; c Institute of Clinical Research, National Hospital Organization, Kure Medical Center, 3-1 Aoyama-cho, Kure, Hiroshima 737-0023, Japan

## Abstract

**Background:**

Liaison critical paths (LCPs) for coronary artery disease (CAD) were developed to support collaborative care for CAD patients between cardiologists in emergency hospitals and referring physicians through sharing of medical information, including cardioprotective medications and cardiovascular risk factors. However, little is known about the effects of LCPs in practice.

**Methods:**

We conducted an observational study of CAD patients undergoing percutaneous coronary intervention in our hospital between September 2007 and June 2010; these patients were managed with an LCP by referring physicians after discharge. We surveyed implementation of scheduled hospital visits, prescription of cardioprotective medicines, and risk factor measurements 6 and 12 months after discharge.

**Results:**

Implementation rate of hospital visits was significantly elevated from 50.7% to 89.3% after establishing LCPs. At the 12-month visit, prescription rates for anti-platelet drugs, statins, β-blockers, and angiotensin-converting enzyme inhibitors or angiotensin II type I receptor blockers were 99.7%, 95.0%, 77.1%, and 74.3%, respectively. Target achievement rates for low-density lipoprotein cholesterol (LDL-C; < 100 mg/dL) and high-density lipoprotein cholesterol (HDL-C; ≥ 40 mg/dL) significantly increased from 48.6% to 64.5% and 62.0% to 82.7%, respectively, while those for body mass index (BMI; < 25 kg/m^2^), blood pressure (< 130/80 mmHg), triglycerides (< 150 mg/dL), and HbA1c (< 7.0 %) were unchanged. BMI, triglycerides, HDL-C, LDL-C, and HbA1c levels significantly improved in patients who implemented all visits. Moreover, risk factor management did not differ significantly between cardiologists and non-cardiologists using LCPs.

**Conclusions:**

LCPs for CAD may facilitate implementation of optimal medical therapy and target achievement of risk factors in practice.

**Keywords:**

Liaison critical path; Coronary artery disease; Cardiovascular prevention; Risk factors; Clinical practice

## Introduction

Current guidelines have documented the significance of systemic therapies that reduce plaque vulnerability through evidence-based use of medication and aggressive intervention for multiple cardiovascular risk factors in reducing the rate of death or myocardial infarction [1-3]. The Clinical Outcomes Utilizing Revascularization and Aggressive Drug Evaluation (COURAGE) trial demonstrated that focal therapy with percutaneous coronary intervention (PCI) for stenotic coronary lesions did not reduce the risk of major cardiovascular events when added to optimal medical therapy (OMT) in patients with stable coronary artery disease (CAD). This provides evidence reinforcing current guidelines supporting the aggressive use of OMT [4]. However, in clinical practice, almost a third of patients are not treated with OMT at discharge following PCI, a pattern that has showed little change even after the publication of the COURAGE trial [5]. These findings indicate that improvements are required in the incorporation of OMT into routine practice.

In Japan, elderly people over the age of 65 years accounted for 21% of the population, the world’s highest, in 2005. This percentage increased to 23.2% in June 2011, and is expected to rise further in the future [6]. To reduce the load on physicians in emergency hospitals, the government has recommended the development of local collaborations between emergency hospitals and general clinics since 2006. According to this policy, recently stabilized patients with CAD tend to be managed by referring primary physicians; however, these doctors are not always familiar with recent evidence for cardiovascular prevention. To resolve this social issue, liaison critical paths (LCPs) for the management of CAD have been developed. Generally, in the LCP system, cardiologists in emergency hospitals and referring physicians cooperatively manage CAD patients using an information tool, i.e., printed information sheets or electronic files on the internet, to share medical information, including severity of CAD, coronary intervention, medications, cardiovascular risk factors, guideline-based targets for the control of risk factors, and schedules of follow-up examinations. Our hospital is in Kure City, where the elderly account for 28.2% of the population [7]; this was Japan’s highest elderly ratio in cities with a population of over 150,000 people in 2010. A hospital-based LCP system for CAD has been established in our hospital since 2007. Recently, in many medical areas, public health centers or medical associations have developed community-based LCPs for CAD, although the management systems and/or information tools are somewhat different among medical areas. However, little is known about the clinical benefits of LCP in practice.

To assess whether LCP for CAD may have the potential to facilitate the implementation of OMT, we surveyed the implementation rate of scheduled hospital visits, the prescription rate of cardioprotective medications, and the achievement rate of guideline targets for risk factors in patients managed with LCP for CAD before discharge and during 1 year of observation. In addition, we compared the management of risk factors by referring physicians using the LCP system between cardiologists and non-cardiologists.

## Methods

### Management system using liaison critical path for CAD

We established an LCP system for the management of patients with CAD in routine practice. This system was introduced to all patients with CAD undergoing PCI who received outpatient treatment in general clinics after discharge. Using this system, all patients received multidisciplinary education on preventing cardiovascular events before discharge, including information on their cardiovascular risk factors, the purpose and effects of their medication, and how to change their lifestyle, i.e., how to make healthy food choices and do suitable exercise. All of this information was described on the LCP information sheet. Furthermore, the LCP information sheet provided each patient’s personal medical information to referring physicians in general clinics, including severity of CAD, coronary intervention, medications, cardiovascular risk factors, the guideline targets for risk factors, and schedules of follow-up examinations in our hospital. Official staff assigned to local clinical liaison sent a list of patients to referring physicians by fax or e-mail to remind them which patients were scheduled to visit our hospital before the scheduled follow-up date. Trained nurses explained the follow-up examinations to patients before the scheduled date by phone. Referring physicians marked checkboxes indicating achievement of guideline targets for each risk factor on the LCP information sheet. In cases where referring physicians suspected progression of angina pectoris or occurrence of cardiac events, they could immediately consult cardiologists in our hospital at any time. Cardiologists in our hospital provided information on the results of follow-up examinations and suggestions for suitable medications to referring physicians.

### Subjects and data collection

The subjects in this study were a consecutive series of 318 patients with CAD undergoing PCI from September 2007 to June 2010 who received outpatient treatment in general clinics with LCP after discharge. In addition, as a control, we surveyed 140 consecutive patients with CAD undergoing PCI from January to December in 2006 who received outpatient treatment in general clinics before the establishment of LCP. The data were collected by retrospectively reviewing all individual medical records during hospitalization and follow-up visits. The first and second visits were scheduled at 6 ± 3 months and 12 ± 3 months after hospital discharge, respectively. The data collected included body mass index (BMI), blood pressure, prescription of several classes of medicines including anti-platelet agents, statins, β-blockers, angiotensin-converting enzyme inhibitors (ACE-Is) or angiotensin II type I receptor blockers (ARBs), and calcium channel blockers (CCBs), and circulating levels of various parameters including total cholesterol, triglycerides, high-density lipoprotein cholesterol (HDL-C), low-density lipoprotein cholesterol (LDL-C), and HbA1c. This study was approved by the Ethics Committee of Kure Medical Center.

### Laboratory measurements

Venous blood was drawn from all of the subjects after an overnight fast. The serum was immediately subjected to laboratory measurements. Serum concentrations of total cholesterol, triglycerides, and HDL-C were determined by enzymatic methods (Sekisui Medical Co., Tokyo). The concentration of HbA1c (JDS: Japan Diabetic Society) was measured by high performance liquid chromatography (TOSOH Co., Tokyo) and then was calculated as HbA1c (NGSP: National Glycohemoglobin Standardization Program) [8]. LDL-C was calculated by the Friedewald formula.

### Achievement of guideline targets for cardiovascular risk factors

We used the following targets for management of cardiovascular risk factors in patients with CAD, according to Japanese guidelines [2, 3, 8-11]: BMI < 25 kg/m^2^, blood pressure < 130/80 mmHg, triglycerides < 150 mg/dL, HbA1c (NGSP) < 7.0 %, LDL-C < 100 mg/dL, and HDL-C ≥ 40 mg/dL.

### Statistical analysis

Data are expressed as mean ± SEM. Paired t-tests were performed to evaluate changes in various parameters after hospital discharge. The chi-square test was performed to assess implementation of scheduled visits, cardioprotective medications, achievement of guideline targets for risk factors, and comparison of medical management between cardiologists and non-cardiologists. All statistical analyses were performed using JMP for Windows software (version 8; SAS Institute, Cary, NC). Statistical significance was defined as a P value of < 0.05.

## Results

### Implementation of scheduled follow-up visits after hospital discharge

There was no significant difference in the baseline characteristics of the patients between before and after establishment of LCP, except HbA1c (Table 1). Table 2 shows the proportion of patients who implemented the hospital visits scheduled at 6 and 12 months after discharge, which significantly increased from 50.7% to 89.3% after establishing LCP.

**Table 1:** Baseline Characteristics of the Subjects

	Without LCP	With LCP	P^#^
	(Jan.-Dec. 2006)	(Oct. 2007-Jun. 2010)	
Number of patients	140	318	
Age (y)	70.8 ± 9.3	69.2 ± 9.9	0.093
Male (%)	66.4	74.2	0.088*
Coronary artery disease			
AMI (%)	29.3	28.9	0.939*
Others (%)	70.7	71.1	
BMI (kg/m^2^)	23.3 ± 3.7	24.1 ± 4.0	0.089
Blood Pressure			
Systolic (mmHg)	133.4 ± 25.3	129.4 ± 20.1	0.058
Diastolic (mmHg)	72.6 ± 15.4	72.8 ± 12.7	0.999
Laboratory			
Triglycerides (mg/dL)	118.5 ± 79.3	125.7 ± 72.2	0.357
HDL-C (mg/dL)	44.4 ± 13.1	44.4 ± 11.7	0.900
LDL-C (mg/dL)	100.7 ± 32.3	104.1 ± 30.3	0.291
HbA1c (%)	6.7 ± 1.6	6.3 ± 1.3	< 0.001

^#^ Statistical analyses were performed by Student’s t-test or chi-square test (*). LCP, liaison critical path; AMI, acute myocardial infarction; BMI, body mass index; HDL-C, high-density lipoprotein cholesterol; LDL-C, low density lipoprotein cholesterol.

**Table 2:** Implementation of Follow-up Hospital Visits With or Without Liaison Critical Path

	Without LCP	With LCP	P^#^
	(Jan.-Dec. 2006)	(Oct. 2007-Jun. 2010)	
Total subjects, n	140	318	
6-month visit, n (%)	75 (53.6)	280 (88.1)	< 0.001
12-month visit, n (%)	71 (50.7)	284 (89.3)	< 0.001

^#^ Statistical analyses were performed by chi-square test. LCP, liaison critical path.

Among the patients managed with LCP, 10.7% did not visit our hospital at 12 months after discharge, which included the 6.6% of patients who gave up the scheduled hospital visits for unavoidable reasons, such as death, muscular neurological disorders, and advanced cancers. Only 4.1% of patients did not implement scheduled visits for other reasons, such as low compliance (data not shown).

### Cardioprotective medications in clinical practice with LCP

**Figure 1:**
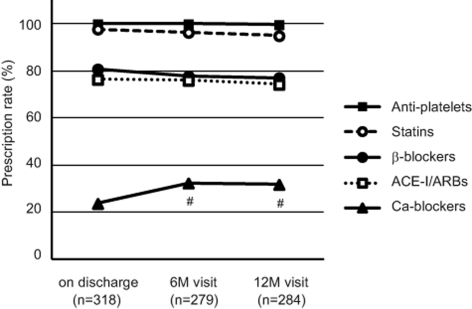
Prescription of several classes of cardioprotective medicines in clinical practice with liaison critical path. Proportions of the patients who were prescribed anti-platelet agents, statins, β-blockers, angiotensin-converting enzyme inhibitors (ACE-Is)/angiotensin II type I receptor blockers (ARBs), and calcium channel (Ca) blockers are shown at indicated times. Six-month (6M) and 12-month (12M) visits were scheduled at 6 ± 3 months and 12 ± 3 months after hospital discharge, respectively. Statistical analyses were performed by chi-square test. #: P < 0.05 vs. on discharge.

Initial prescription rates for anti-platelet agents, statins, β-blockers, and ACE-Is or ARBs were very high (100.0%, 97.8%, 80.8%, and 76.4% on discharge, respectively). This high prescription rate was maintained during the follow-up period (99.7%, 95.0%, 77.1%, and 74.3% at the 12-month visit, respectively; Fig. 1). The prescription rate for CCBs increased from 23.6% on discharge to 32.3% and 31.7% at the 6- and 12-month visits, respectively (both P < 0.05 vs. on discharge; Fig. 1).

### Achievement of guideline targets for risk factors in clinical practice with LCP

**Figure 2:**
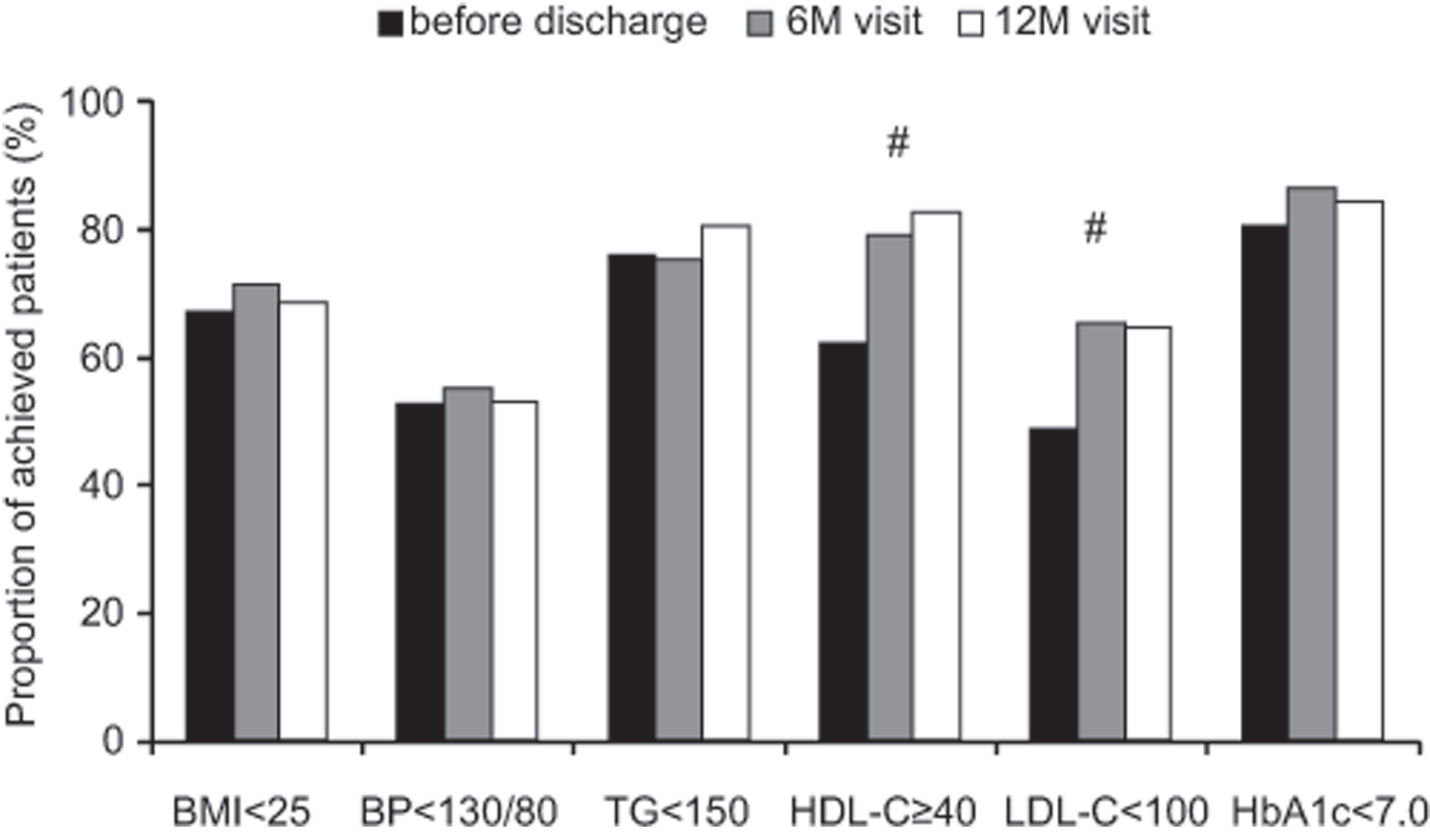
Achievement rates of guideline targets for cardiovascular risk factors in clinical practice with liaison critical path. Proportions of the patients who achieved body mass index (BMI) < 25 kg/m^2^, blood pressure (BP) < 130/80 mmHg, triglycerides (TG) < 150 mg/dL, HbA1c < 7.0 %, low density lipoprotein cholesterol (LDL-C) < 100 mg/dL, and high density lipoprotein cholesterol (HDL-C) ≥ 40 mg/dL are shown at indicated times. Six-month (6M) and 12-month (12M) visits were scheduled at 6 ± 3 months and 12 ± 3 months after hospital discharge, respectively. Statistical analyses were performed by chi-square test. #: P < 0.05 vs. before discharge.

The achievement rate for LDL-C < 100 mg/dL showed a significant increase from 48.6% before discharge to 64.5% at the 12-month visit (P < 0.05), and that for HDL-C ≥ 40 mg/dL also significantly increased from 62.0% before discharge to 82.7% at the 12-month visit (P < 0.05; Fig. 2). Achievement rates for triglycerides < 150 mg/dL and HbA1c < 7.0% were 80.5% and 84.5% at the 12-month visit, respectively; these rates were high before discharge and were maintained over 1 year (Fig. 2). On the other hand, the achievement rates for BMI < 25 kg/m^2^ and blood pressure < 130/80 mmHg were 68.6% and 53.1% at the 12-month visit; these rates were not improved during 1 year of observation (Fig. 2).

### Control of cardiovascular risk factors in clinical practice with LCP

**Figure 3:**
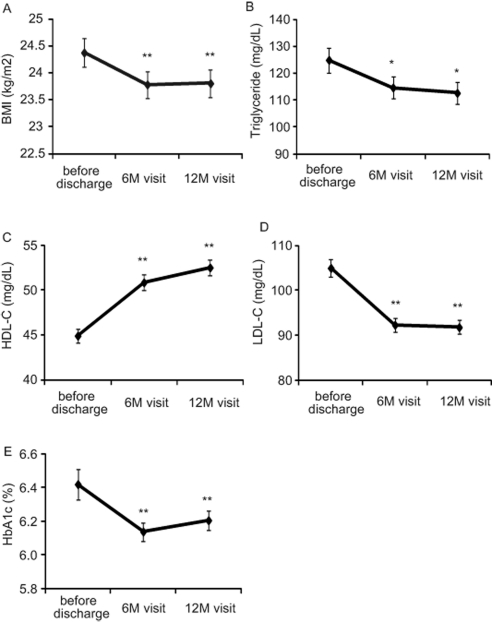
Changes of several parameters associated with risk factors in clinical practice with liaison critical path. Body mass index (BMI) (kg/m^2^) (A), triglycerides (mg/dL) (B), high density lipoprotein cholesterol (HDL-C) (mg/dL) (C), low density lipoprotein cholesterol LDL-C (mg/dL) (D), and HbA1c (%) (E) are shown at indicated times. Six-month (6M) and 12-month (12M) visits were scheduled at 6 ± 3 months and 12 ± 3 months after hospital discharge, respectively. Data are expressed as mean ± SEM. Statistical analyses were performed by paired t-test. **: P < 0.001, *: P < 0.01 vs. before discharge.

In the patients who implemented all visits, BMI, LDL-C, HDL-C, and triglyceride levels were significantly improved over the follow-up period (BMI, P < 0.001; LDL-C, P < 0.001; HDL-C, P < 0.001; triglycerides, P < 0.01; Fig. 3). HbA1c significantly decreased at the 6-month visit (P < 0.05), but this was slightly reversed at the 12-month visit (Fig. 3).

### Management of CAD patients with LCP by cardiologists and non-cardiologists

Next, we compared the management of CAD patients with LCP by referring physicians between cardiologists and non-cardiologists. As shown in Table 3, there was no significant difference in the proportion of patients who visited our hospital for follow-up according to the LCP schedule between those treated by cardiologists and those treated by non-cardiologists. Moreover, the achievement rates of the guideline targets for risk factors at the 12-month visit did not differ significantly between cardiologists and non-cardiologists (Table 3).

**Table 3:** Management of Coronary Artery Disease Patients by Cardiologists and Non-Cardiologists With Liaison Critical Path

	Cardiologist	Non-cardiologist	P^#^
	(n = 45)	(n = 74)	
Implementation of scheduled visit			
6-month visit, (%)	86.2	89.4	0.386
12-month visit, (%)	85.4	92.0	0.060
Achievement of targets (12-month)			
BMI < 25 kg/m^2^, (%)	72.1	66.3	0.307
Blood pressure < 130/80 mmHg, (%)	51.4	55.7	0.482
Triglycerides < 150 mg/dL, (%)	80.0	80.8	0.865
HDL-C ≥ 40 mg/dL, (%)	80.8	83.9	0.503
LDL-C < 100 mg/dL, (%)	69.2	61.3	0.185
HbA1c < 7.0 %, (%)	88.8	81.8	0.123

^#^ Statistical analyses were performed by chi-square test. BMI, body mass index; HDL-C, high-density lipoprotein cholesterol; LDL-C, low density lipoprotein cholesterol.

## Discussion

Our study demonstrates that the proportion of patients implementing follow-up hospital visits was significantly elevated after development of an LCP system. The prescription rates of cardioprotective medicines including anti-platelet agents, statins, ACE-Is or ARBs, and β-blockers were very high on discharge after PCI, and these rates were maintained over 1 year. The proportion of patients who achieved a LDL-C level < 100 mg/dL and a HDL-C level ≥ 40 mg/dL was significantly elevated during 1 year of observation. BMI, triglycerides, HDL-C, LDL-C, and HbA1c were significantly improved in the patients who implemented all visits scheduled by the LCP. Moreover, our study demonstrated that there was no significant difference in the management of CAD patients between cardiologists and non-cardiologists when using LCP, suggesting that LCP may be an effective way to bridge the gap between published evidence-based guidelines and routine clinical practice.

Despite the establishment of evidence-based guidelines, prevention therapies are sometimes underused in daily clinical practice [5, 12-14]. Borden, et al. reported that even after the publication of the COURAGE trial, prescription rates of statins, ACE-Is or ARBs, and β-blockers were 84.7%, 60.7%, and 75.9%, respectively, for patients after PCI [5]. In our study, the prescription rates of these medicines were 95.0%, 74.3%, and 77.1% at 1 year after PCI, respectively, in patients treated by general primary physicians with LCP. This suggests that the LCP system may support general primary physicians to facilitate the implementation of evidence-based medication for cardiovascular prevention.

During clinical practice by Japanese general physicians, the achievement rates of guideline targets for LDL-C and systolic blood pressure have been reported to be 57.1% and 34.0%, respectively, although the subjects in this study included patients without CAD [15]. In our survey, subjects managed with LCP showed relatively higher achievement rates of guideline targets; those for LDL-C and blood pressure were 64.5%, 53.1%, respectively. This suggests that the LCP system may increase the opportunity for general primary physicians to develop their awareness of the achievement of guideline targets. Patients who achieve guideline targets show significantly less cardiovascular events than those who do not [15]. Therefore, it can be expected that the LCP system used in the current study may be effective for suppression of cardiovascular events, although we did not investigate cardiovascular events directly.

Thus, the LCP system used seems to be effective in the implementation of guideline-based strategies for cardiovascular prevention. This may be not only due to sharing of medical information between emergency hospitals and general clinics but also due to the multidisciplinary education on cardiovascular prevention received by the patients. Such education helps patients to understand the significance of controlling their cardiovascular risk factors, learn about the purpose and effects of their medication, and be more motivated to change their lifestyle. In addition, another essential component of the LCP system used may be the reminders for follow-up hospital visits sent to both the referring physicians and the patients before scheduled examinations. In turn, this LCP system should increase the opportunity for cardiologists who are aware of up-to-date evidence to conduct optimal medical control of risk factors.

Nevertheless, in our study, the continuous control of body weight, blood pressure, and diabetes seemed to be difficult even when using the LCP system, which suggests that continuing a healthy life-style is more difficult for patients than merely starting one. Our LCP system seems to be less effective for continuous improvements in lifestyle for patients. Another system is required to continuously motivate patients to maintain a healthy life-style.

Our study provides suggestive data on the effects of LCP but not conclusive evidence, as it was an observational study based on retrospective investigation of medical records in a single center. To further investigate the effectiveness of LCP systems, including effects on prevention of cardiovascular events, multi-center prospective studies with larger samples are required.

We live in an aged society, where even high-risk patients should be managed in practice by general physicians, rather than cardiologists. In addition, implementation of evidence-based cardiovascular prevention strategies in practice is becoming more important. Our data suggest that LCP for CAD may have the potential to facilitate implementation of evidence-based medicine in practice, although further investigations are required.
